# Change of Rice Paddy and Its Impact on Human Well-Being from the Perspective of Land Surface Temperature in the Northeastern Sanjiang Plain of China

**DOI:** 10.3390/ijerph19159690

**Published:** 2022-08-06

**Authors:** Tao Pan, Zhengyi Bao, Letian Ning, Siqin Tong

**Affiliations:** 1 School of Geography and Tourism, Qufu Normal University, Rizhao 276826, China; 2 Key Laboratory of Land Surface Pattern and Simulation, Institute of Geographic Sciences and Natural Resources Research, Chinese Academy of Sciences, Beijing 100101, China; 3 College of Computer Science and Technology, Inner Mongolian Normal University, Hohhot 010070, China; 4 College of Geographical Science, Inner Mongolia Normal University, Hohhot 010022, China

**Keywords:** land use change, paddy field, land surface temperature, Sanjiang Plain of China

## Abstract

Large-scale and high-speed paddy land expansion has appeared in Northeast China since the 21st century, causing the change in land surface temperature. The lack of continuous investigation limits the exploration of discoveries in this region. To address this limitation, a collaborative approach that combined human–computer interaction technology, gravity center model and spatial analysis was established. It provided some new findings in spatiotemporal evolution, migration trajectory and surface cooling effect of the paddy field in Northeastern Sanjiang Plain, a center of paddy field planting in China. The results show that: (1) A sustained paddy expansion was monitored, with a total area ranging from 2564.58 km^2^ to 11430.94 km^2^, along with a rate of growth of 345.72% from 2000 to 2020. Correspondingly, its reclamation rate changed to 47.53% from 10.66%, showing the improved planting level of the paddy field. (2) Gravity center of paddy field continued to be revealed northward with a 5-year interval from 2000 to 2020. Migration distance of the straight line reached 23.94 km^2^, with the direction offset of 27.20° from east to north. (3) Throughout the growing season of crops, the land surface temperature of paddy field was 27.73°, 29.38°, 27.01°, 25.62° and 22.97° from May to October; and the cooling temperature effect of paddy field was investigated, with the reduced values of 0.61°, 0.79° and 1.10° in the low-, medium- and high-paddy field density regions from 2000 to 2020, respectively. Overall, these new findings in the cold temperate zone, high latitude region of the Northern Hemisphere, provided the reference for the investigation of paddy field monitoring and its environmental effects in China and other regions.

## 1. Introduction

Monitoring the process of paddy field development level and its dynamic changes was a very significant work for the global population and environmental changes [1], considering that paddy field provided the basic rations for the growth of global population and the environmental issues caused by paddy field itself [2,3,4]. Since 2000, the global population has increased from 6.11 billion to 7.94 billion, along with a total rate of 29.95%, which has required more food and agricultural products [5,6]. The basic ration of rice crops played an important role in this process, and the paddy field planting scale continued to increase. The expansion of paddy fields also affected the issues of the hydrological cycle [7,8], methane emission [9], climate change [10,11], surface radiation energy balance [12] and soil organic matter content. It has always been one of the hot issues in the world to carry out the capture of paddy field dynamic information and the study of its dynamic change and environmental effects.

In China, rice was favored in the food among the Chinese people [13], and paddy fields were planted on a large scale. Traditional paddy field planting was located in the southeast coastal region of China [14,15]. This region belonged to the warm temperate belt and humid zone. Sufficient rain and light provided the convenient conditions for paddy field planting [16,17]. However, the rapid urbanization, industrialization and rural land expansion in southeast coastal China have led to the large-scale loss of traditional paddy fields, causing huge pressure on the continuous and stable supply of paddy rice in China. Then, the planting of paddy fields gradually migrated to the north, from the warm temperate belt to the middle temperate belt, the cold temperate belt [18]. Northeast China lay in the cold temperate zone. The humid climate, abundant water resources, flat terrain, high-quality soil and other natural factors made Northeast China become a grain supply base for China [19,20]. In this process, grain planting in Northeast China was mainly upland crops first. Then, the planting of paddy fields can be gradually increased with the support of a series of water conservancy projects and agricultural policies [21]. In Northeast China, there was a special region named Sanjiang Plain. Paddy field planting in Sanjiang Plain had two advantages when compared with the traditional paddy fields in southeast coastal China. One was the relatively low pressure on construction land. In Sanjiang Plain, urbanization, industrialization and rural land expansion were generally weak compared with southeast coastal areas. This means that paddy fields were facing low loss area and the stability of paddy field planting area was greatly improved, bringing about the continuous production supply of rice paddy. The other was the unique role of grain supply of Sanjiang Plain in China; namely, Sanjiang plain was a national commodity grain production base in China [22]. The grain here was transported to all parts of China, feeding the people. The suitable climate and unique natural endowment made the taste of paddy fields in Sanjiang Plain better, along with the full granules, soft and delicious, which was more suitable for Chinese people’s preference for paddy fields when compared with the traditional paddy fields in southeast coastal China. According to previous literature, paddy field planting in Sanjiang Plain has played a leading role in Northeast China since 2000 [23]. Relying on the advantages of terrain, water resources, agricultural policies and irrigation projects, the Northeastern Sanjiang Plain has become the first region to carry out large-scale development of paddy fields, as well as the loss of wetlands and other lands [23,24]. However, the continuous monitoring of paddy fields in this region is insufficient, which limits the excavation of new findings.

The land surface temperature may be a factor affecting human well-being, and the expansion of paddy fields changed the land surface temperature [25,26]. A characteristic of paddy field growth was that it was transplanted in a flooded environment (i.e., water body and soil environment) [23]. When paddy fields invaded adjacent land use types such as the upland crop region, the waterless environment of upland crops was replaced by the flooded environment of paddy fields, bringing about a change in the balance of surface radiation energy, such as the reduced sensible heat flux and increased latent heat flux [27]. Such energy change may bring comfort to the living environment in agricultural planting regions in hot summer, considering the evaporation and transpiration from the large-scale paddy fields [28,29]. The land surface temperature was a direct indicator reflecting human well-being [30]. Most surveys that study human settlements’ well-being based on land surface temperature focused on urban regions [31,32] due to the impact of urban heat waves and heat island effects on the dense population. However, the relevant investigations in agricultural areas were insufficient. The cooling effect of irrigated farmland in summer even exceeded the warming effect of urban heat islands in some regions of the world [33,34], indicating that the irrigated cultivated land has an important impact on climate on a global scale. Additionally, the cooling effect of irrigated paddy fields may bring physical comfort to agricultural workers or the farmers in the hot summer in the high latitudes of China, serving the human well-being [35], but the continuous follow-up investigation in this region from the paddy field is insufficient, such as the new effects of paddy field on the different months of the growing season and multi-year scale cooling effect value.

To reveal the new change of paddy field expansion and its new environmental effect in 2020. A paddy field planting center of China, the Northeastern Sanjiang Plain, was focused as the study area. Through the latest paddy field data as well as the LST product, we revealed the new changes in reclamation level and the characteristics of the gravity shift of the paddy field. Further, the regional cooling effect brought by paddy field expansion was explored from the perspective of human settlement comfort. Therefore, the research objectives of this paper are: (1) to reveal the quantitative dynamics, reclamation rate and regional differences of paddy fields from 2000 to 2020 and (2) to investigate the spatial evolution of paddy fields and their centers of gravity migration trajectory at 5-year intervals from 2000 to 2020 (i.e., 2000–2005, 2005–2010, 2010–2015 and 2015–2020), and (3) to analyze the different temperature differences of paddy fields in each month during the whole crop growing season, and explore the cooling effect of different paddy field scale areas from 2000 to 2020. In addition, we discussed the findings of this study from the aspect of the new finding of paddy field expansion in the study area from 2000 to 2020, the influence of paddy field expansion on the comfort of human well-being, and other possible environmental effects from paddy field expansion. We expect this study to provide a reference for paddy change and its environmental effect in other regions.

## 2. Method

### 2.1. Study Area

**Figure 1:**
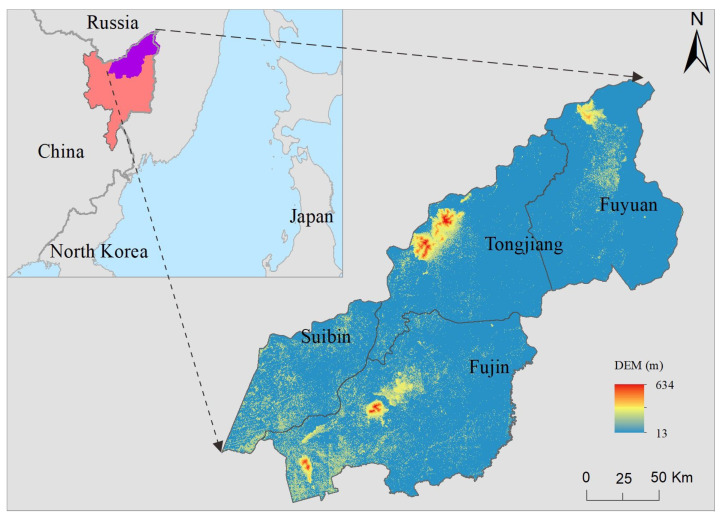
Location map of the study area. Note: In the location diagram in the upper left corner, the red area is Sanjiang Plain, and the purple area is our study area.

Our study area was located in the northeast part of Sanjiang Plain (Figure 1), covering a total area of over 24,000 km^2^. Administrative divisions included 4 cities/counties of Suibin, Fujin, Tongjiang and Fuyuan. The study area was in high latitude, accompanied by the temperate continental monsoon climate. It was cold in winter and hot in summer. The frost-free period was 120~140 days, and the active accumulated temperature above 10 °C was 2300~2500 °C. The annual precipitation was 500~650 mm, and 75~85% was concentrated during June–October (i.e., the crop growing seasons). Although the latitude in the study was high, the summer was warm, and the average temperature of the hottest month was above 22 °C. It is rainy and hot in the same season, which is suitable for the growth of agriculture, especially for the high-quality rice paddy. In the meanwhile, the terrain of the study area was flat, which was very convenient for land leveling and water storage for paddy patches. The content of soil organic matter was high, providing a better soil environment for paddy field planting. The surface water resources were abundant, and the main rivers included the names of Heilongjiang river, Wusuli river, Songhua river and Naoli river. 

### 2.2. Method of the Technical Process

The objectives of this study were to explore the spatiotemporal heterogeneity, migration process, and cooling temperature effect from paddy field expansion. The human–computer interaction technology was applied to obtain land use in 2015 and 2020, extending the land use time node from 2000 to 2020 and providing the paddy land for spatiotemporal heterogeneity investigation. Then, the gravity center model was used to track the process of paddy field migration and to understand the migration distance and direction of paddy field energy. Spatial processing technology and mathematical analysis were applied to reveal the cooling temperature effect of paddy fields. Overall, a collaborative approach that combined the human–computer interaction technology, gravity center model and spatial analysis was established to gradually complete the research objectives of this study. 

**Figure 2:**
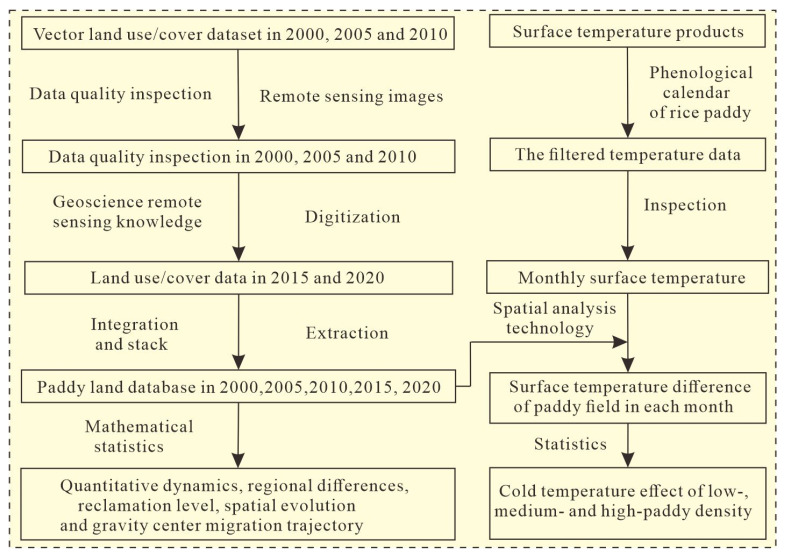
The mainly technical process of spatiotemporal monitoring of paddy field and its impact on surface temperature from 2000 to 2020.

We collected land use data, surface temperature data and auxiliary data. For land use data, because the collected land use data only had three periods of 2000, 2005 and 2010, human–computer interaction technology was used to obtain land use data in 2015 and 2020. In this method, taking the remote sensing images as the background maps, we superimposed the vector land use data onto these images and manually interpreted the vector dynamic maps of each land use type using professional geoscience knowledge through the different land use features such as shape, texture, color, size and aggregation state from remote sensing images. The advantage of this method was that it can eliminate the influence of spectral differences on data classification (i.e., different objects had the same spectrum and the same object had different spectrums) and effectively improve data accuracy. After accuracy evaluation, the quantitative dynamics, regional differences, reclamation level and spatial evolution were analyzed at 5-year intervals from 2000 to 2020. Then, gravity center model was applied to reveal the geographic coordinates, distance and direction of the paddy field migration process. After that, using the spatial data processing and statistical analysis, the together of land use data and land surface temperature data was applied to explore the monthly land surface temperature differences of paddy field across the growing seasons and the different temperature cooling effects of low-, medium- and high-density paddy fields. The main technical process of this study is provided in the below figure (Figure 2).

### 2.3. Data Collection

The data that we used in this study mainly included land use data, land surface temperature data and auxiliary data. Specifically, the land data for 2000, 2005 and 2010 were from the Institute of Geographical Sciences and Resources, CAS, and the data format was vector that can obtain more accurate statistical results and spatial patterns. The land surface temperature data was from NASA’s official website. The auxiliary data mainly consisted of Landsat TM/ETM+/OLI, high-resolution google images, Gao-fen series satellites, digital elevation model, administrative division data and river distribution map. 

### 2.4. Processing and Visualization of Land Use Data to Extend to 2000–2020

The processing of land use data mainly included two aspects: the first was the inspection of the data that were already obtained in 2000, 2005 and 2010, and the second was the production of data in 2015 and 2020, respectively. For the visual inspection of the obtained data [36], we needed the remote sensing image of the natural background as the foundation and downloaded the images of the corresponding year through the USGS official website. The time selection of images was mainly focused on the growing season of crops, especially in late spring (i.e., May and June). During this period, rice paddy was in the transplanting stage (i.e., the process of transplanting rice seedlings from seedling sheds to field plots in Northeast China). The surface coverage of paddy fields was dominated by the combination of water and seedlings during this period [23]. Correspondingly, the remote sensing images of paddy fields showed blue regularly shaped spots, which was conducive to checking the quality of paddy fields. We synthesized these downloaded images in a true color approach. Such a synthesis method conveniently presented the real color of the land surface [37]. Through professional geography and remote sensing knowledge, we superimposed the vector land use data on the corresponding remote sensing image for quality inspection and correction to ensure the high accuracy and high quality of land use data in 2000, 2005 and 2010.

Then, according to the vector land use data in 2010, we superimposed it on the remote sensing images in 2015 in the professional geographic information system data processing platform. A manual digitization method was implemented to obtain the vector dynamic data in the period of 2015–2020. The feature identifications of land use data in remote sensing images, such as the outline of the patches, the presented color, the land cover texture, the scale and size, and the spatial distribution morphology, were fully considered when we identified the change patches of each land use type. Considering that the manual digitization was vulnerable to human factors, we used different people to obtain the secondary dynamic land use patches in the same regions to reduce the impact of different professional knowledge backgrounds from the persons on the land use dynamic map. Similarly, the dynamic map of land use from 2015 to 2020 was obtained in the same way. After that, stratified random sampling was applied for accuracy evaluation, and the results were all well, indicating that the land use data were credible and could be used in this study. 

### 2.5. Center of Gravity Transfer Model

The ordinary analysis of spatiotemporal characteristics of paddy fields can only describe the number and spatial pattern changes of paddy fields, but it cannot reveal the overall migration process of paddy fields, such as migration distance and migration direction. In Sanjiang Plain, the cold temperate zone of the high latitudes in the Northern Hemisphere, the migration process of paddy fields, such as going north, overcoming low temperatures and cold waves and providing food for mankind, had important practical significance and served food security. To reveal this process, the center of gravity migration model was used in this study. The advantage of the gravity model was that it could effectively reveal the geographical coordinates, distance and direction of paddy field changes and accurately track the process of paddy field migration. We used the center of the gravity model to calculate the transfer process of paddy field change, to provide the continuous change in geographical coordinates, direction and distance, with a 5-year interval from 2000 to 2020. The geographical coordinates of the center of gravity of the surface temperature were obtained by Equation (1). The corresponding moving direction and distance were calculated through Equations (2) and (3). *R* was a constant value, taken at 111.111. The calculation principle of Equations (1)–(3) is provided below [38,39].

(1) $$\bar{x}\;=\;\frac{\sum_{i}^{n}m_{i}y_{i}}{\sum_{i}^{n}m_{i}},\bar{y}=\frac{\sum_{i}^{n}m_{i}y_{i}}{\sum_{i}^{n}m_{i}}$$

(2) $$\theta_{i-j}=\frac{n\pi }{2}+arctg(\frac{y_{i}-y_{j}}{x_{i}-x_{j}})$$

(3) $$D_{i-j}=R\times \sqrt{{\left(y_{i}-y_{j}\right)}^{2}+{\left(x_{i}-x_{j}\right)}^{2}}$$

### 2.6. Processing of Land Surface Temperature Data

Moderate Resolution Imaging Spectroradiometer (i.e., abbreviation: MODIS) was hung on the satellite named Terra, which observed the earth’s surface once every 1–2 days at a medium resolution level (0.25~1 km) in 36 mutually registered spectral bands to obtain the images of land and ocean temperatures, primary productivity, land surface cover, clouds, aerosols, water vapor, fire and other productions. MODIS products have been widely used in scientific research and related investigations and achieved good experimental results, such as the land surface temperature product [40,41]. MODIS’ land surface temperature (LST) products mainly included two time resolutions, namely, the 1-day (i.e., daily LST product) and 8-day (i.e., the synthetic LST product). The daily LST product was obtained by covering the global surface every day from the Terra satellite. Due to natural environmental factors, such as rain, hurricane, cloud cover, etc., the map of daily LST products was always prone to bad pixels. The 8-day LST product was synthesized according to the 8-day daily land surface temperature data. Such a synthesis product can effectively avoid the impact of bad weather on the surface temperature and improve the effectiveness of temperature observation. This product was thus widely used for land surface temperature observation, investigation and research at regional and global scales. Our study used an 8-day MODIS land surface temperature product, which was downloaded from the NASA official website (https://ladsweb.modaps.eosdis.nasa.gov/search/order/3/MOD16A2–6/, accessed on 1 July 2021). Through the paddy phenological calendar in Northeast China, data on crop growing season (i.e., the months of May to October each year) were downloaded. We visually checked all the data and fixed possible bad pixels using the aunspline approach. Then, the land surface temperature data were analyzed by spatial analysis and mathematical statistics with the paddy land data. 

In this part, we investigated the relationship between different paddy field sizes and land surface temperature. To achieve the spatial matching of datasets from different sources (i.e., paddy land dataset and land surface temperature dataset), both datasets were processed into 1984 coordinates and Albers projection. After the same projection and coordinate, we processed the dataset in the same format. The vector format of the paddy land dataset was aggregated into the TIF format of grids. In this process, we set the size of the grid pixel to 1 km × 1 km resolution. On each 1 km grid, the area ratio of paddy field to pixel formed the paddy field density value in the corresponding pixel on the geographic information system data processing platform. We performed the density mapping on all vector paddy field datasets to obtain the paddy field map with the ratio of paddy field in each pixel (i.e., 1 km × 1 km) from 0.01% to 100%. After that, we checked the quality of the 1 km grid land surface temperature dataset to ensure that all pixels had the values and there was no vacancy. Then, the paddy field dataset and land surface temperature dataset were superimposed under the same projection, coordinate and spatial resolution. We visually checked the spatial pixels of these two datasets to ensure that they were spatially matched in each grid. The advantage of spatial data processing was that it could ensure that data from different sources became the same projection, coordinate and spatial resolution so that all kinds of data can be perfectly matched. 

Then, the trisection method was implemented to divide the paddy field density map into low-density (i.e., from 0.01% to 33.33%) regions, medium-density (i.e., from 33.34% to 66.66%) regions and high-density (i.e., from 66.67% to 100.00%) regions. The purpose of dividing different paddy field planting ranks was to distinguish the planting scale of paddy fields per unit area so as to carry out the surface temperature survey in the study. 

## 3. Results

### 3.1. Change Analysis of Paddy Field Quantity and Its Difference in Different Regions

#### 3.1.1. Analysis of Paddy Field Change and Its Reclamation Rate from 2000 to 2020

The total area of paddy field increased from 2564.58 km^2^ in the year 2000 to 11430.94 km^2^ in the year 2020 in the study area, with a total rate of growth of 345.72% and an average annual growth rate of 17.29%. From the characteristics of paddy field change with a time interval of 5 years during 2000–2020, the net change of paddy field area was 1446.74 km^2^, 3647.41 km^2^, 3519.53 km^2^ and 252.68 km^2^ in the period of 2000–2005, 2005–2010, 2010–2015 and 2015–2020, respectively. The data showed that the fastest increment of paddy fields occurred in 2005–2010.

**Figure 3:**
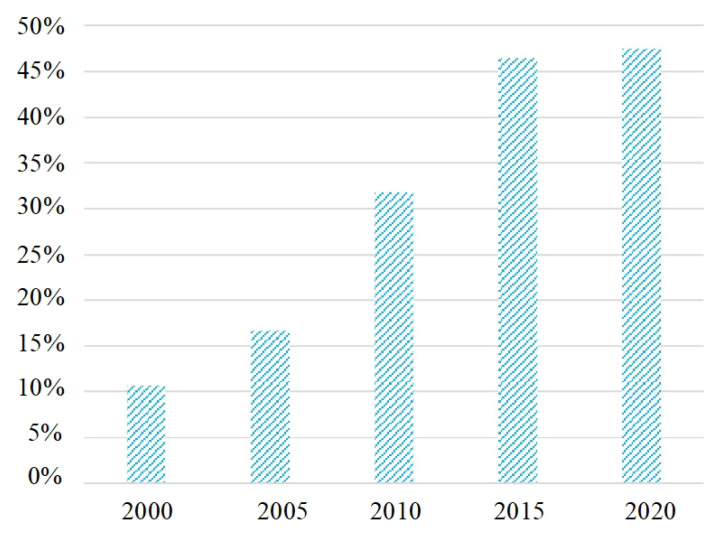
Reclamation rate of paddy land with 5-year interval from 2000 to 2020 in the study area.

Reclamation rate was an important indicator of the proportion of paddy fields in its administrative divisions, which was often used to measure the development level of paddy fields in a region. In 2000, the reclamation rate of paddy fields in the study area was only 10.66%, indicating that the development level of paddy fields was very low. Perhaps, it is in the initial stage of paddy field development. Then, a continuous and intense paddy field development process began to appear, which effectively improved the development level of paddy fields, with the reclamation rate of 16.68%, 31.85% and 46.48% in 2005, 2010 and 2015, respectively (Figure 3). Then, paddy field development began to enter a slow stage, with a reclamation rate of 47.53% in 2020. The data showed that from 2015 to 2020, the increment of paddy field reclamation rate was only 1.05%. Therefore, paddy field development experienced from the initial stage, rapid development stage, and slow stage in the period of 2000–2005, 2005–2015 and 2015–2020. In this process, the development level of the paddy field continued to improve.

#### 3.1.2. Analysis of Regional Differences in the Development of Different Paddy Field Levels from 2000 to 2020 in the Study Area

Analyzing the change process of paddy field reclamation rate in different regions can effectively understand the development level of paddy fields in different regions. In 2000, paddy field reclamation rates in different regions were generally at a low level. Specifically, the lowest value appeared in Fuyuan, with only the reclamation rate of 3.67%, and the maximum value appeared in Suibin, with the corresponding value of 15.71%. Tongjiang and Fujin were among them, namely, 9.60% and 14.45%, respectively. Then, the rapid and intense paddy field expansion happened in each region. In the ending year, the reclamation rates in Fuyuan, Tongjiang, Suibin and Fujin were 50.95%, 42.08 %, 52.78 % and 46.92 %. Data showed that the reclamation rate of Suibin was the highest not only in the initial year but also in the final year. The lowest value of paddy field reclamation rate appeared in Tongjiang in 2020. Although the different regions experienced an improvement in paddy field development level from 2000 to 2020, the fastest development of paddy fields appears in Fuyuan, where the paddy field reclamation rate was the lowest in the initial year. Fuyuan obtained a paddy field reclamation rat +47.28% (i.e., from 3.67% to 50.95), followed by Suibin (+37.07%), Tongjiang (+32.48%) and Fujian (+32.47), respectively (Table 1). In 2020, the reclamation rate of two regions exceeded half, namely, the Fuyuan and Suibin. 

### 3.2. Spatial Heterogeneity and Center Migration of Paddy Field Change from 2000 to 2020

#### 3.2.1. Spatial Pattern of Paddy Field Change from 2000 to 2020 in the Study Area

**Figure 4:**
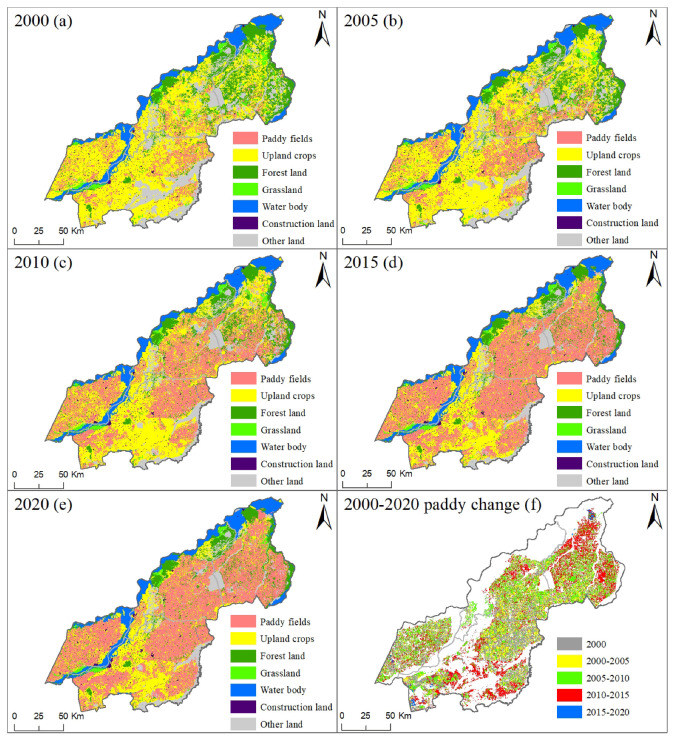
The spatial maps of paddy fields in the years 2000, 2005, 2010, 2015 and 2020 and their change with 5-year intervals from 2000–2020.

The description of the paddy field spatial pattern provided the convenience for understanding the development process of the paddy field in the study area. In 2000 (Figure 4), the planting of paddy crops was mainly distributed in the regions of the central region of Suibin, the southwest and northeast regions of Fujin, and the southern regions of Tongjiang and Fuyuan. Then, the development of the paddy field expanded significantly to other spaces in a small range. An interesting phenomenon was that the original paddy fields were basically reserved. Centered on these reserved paddy fields, paddy fields began to encroach on other surrounding land use types from 2000 to 2005. Furthermore, paddy fields expanded on a large scale and violently towards convenient areas for surface water resources irrigation, such as rivers and their tributaries. These areas provided an important basis for paddy field artificial irrigation from 2005 to 2010. Then, the large-scale expansion of paddy fields continued in the period of 2010–2015. At this stage, as the land near the rivers was already basically reclaimed into paddy fields, paddy fields began to expand away from surface rivers with the support of groundwater irrigation and agricultural irrigation projects. The centralized planting pattern of paddy fields has been widely formed in the regions of Fuyuan, Tongjiang, Suibin and Fujin. In the last period (i.e., 2015–2020), most of the paddy field suitable areas have been developed, considering that the comprehensive impact came from water resources, water conservancy projects, soil conditions and terrain flatness. At this stage, the expansion of paddy fields was mainly concentrated in the southwest part of Suibin and Fujin and the region of northern Tongjiang and Fuyuan. Overall, paddy fields showed complex spatial variation characteristics in each period with a 5-year interval from 2000 to 2020.

#### 3.2.2. Analysis of the Center Migration Process of Paddy Field Change

**Figure 5:**
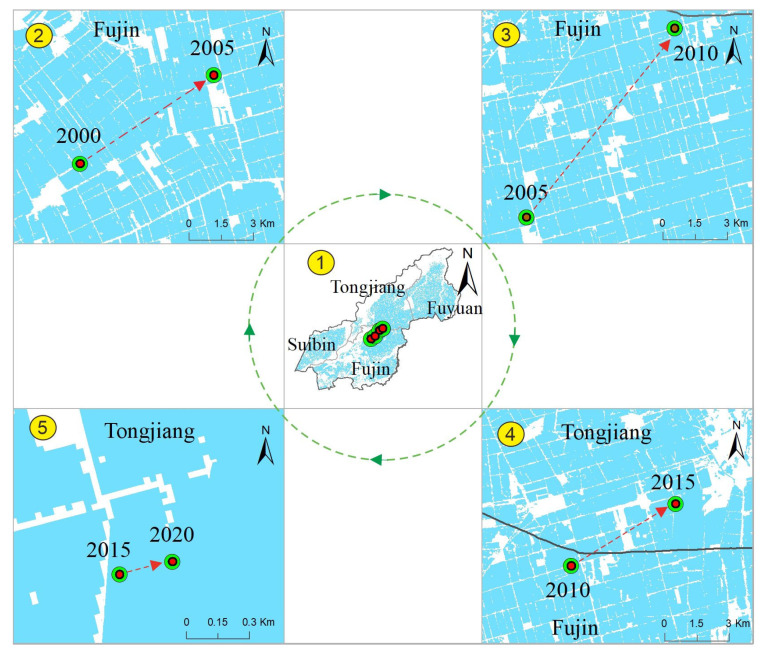
Center of gravity migration trajectory from 2000 to 2020. Note: number 1 was the study area, and number 2 to 5 was the center of gravity transfer with 5-year interval from 2000–2020.

Calculating the spatial position and change process of the center of gravity of the paddy field at each research time node can clearly clarify the process of paddy field migration, which was conducive to understanding the surface radiation energy balance index with the paddy field as the carrier, such as the overall migration of surface temperature, to serve the comfort of human settlements, due to the condition that the migration of gravity center was often accompanied by the synchronous migration of surface temperature. In 2000 (Figure 5), the coordinates of the center of gravity of the paddy field were 132°47′04″ E and 47°25′13″ where located in the northeast part of Fujin city. Then, from 2000 to 2005, the length distance of the center of gravity migration was 7483.46 m, with the 36.24° direction offset from west to north. It suggested that the energy carried by paddy fields, such as surface temperature, migrated in this direction as a whole. From 2005 to 2010, a longer migration distance appeared than in the previous period. Through the calculation, the migration distance was 11,213.85 m. Additionally, the direction of offset was larger, with a value of 52.17°. The center of this period was still located in the northeast part of Fujin City, but it was very close to the boundary between Fujin and Tongjiang. From 2010 to 2015, the overall migration direction of the center of gravity was similar to the one before, and the distance was 5659.64 m. At this stage, the center of gravity has moved from Fujin city to Tongjiang City, where the boundary distance between them was 2188.90 m. From 2015 to 2020, the migration distance of the paddy field gravity center was only 302.72 m, along with the direction of 19.76° from the west to north, in the south region of Tongjiang city. Over, the center of gravity of the paddy field in the study area experienced a complex migration process, with a distance in a straight line of 23,942.87 m from the first point to the last point and the direction offset of 27.20° from east to north. The center of gravity also moved from Fujin city to Tongjiang City.

### 3.3. Analysis of Surface Temperature Change Process of Paddy Field in the Whole Growing Season from 2000 to 2020

**Figure 6:**
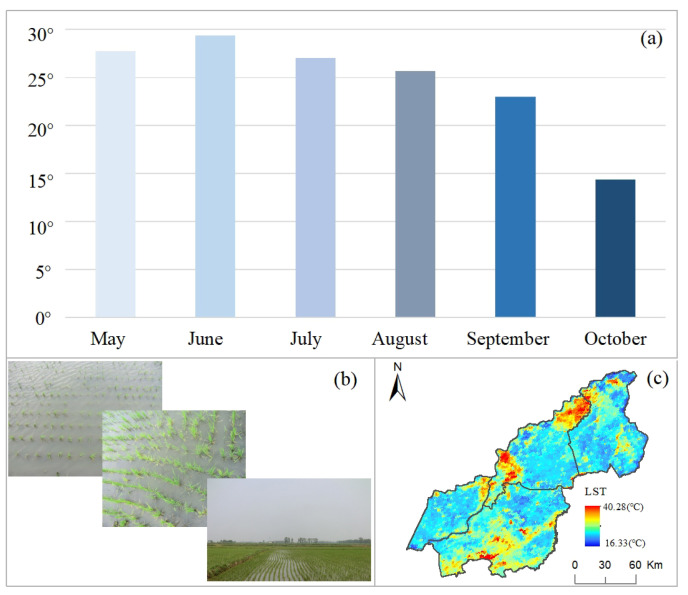
Land surface temperature of paddy field across the growing seasons from 2000 to 2020. Note: (**a**) the average temperature from a paddy field each month across the growing season, (**b**) field investigation of paddy field in the study area and (**c**) spatial pattern of land surface temperature in July of the study area.

According to the crop phenological calendar in Northeast China, crops were ripe once a year due to the winter being cold and the ground being covered with snow. The growing season of crops was concentrated from May to October according to agricultural phenology in the study area. In the first month of crop growth, namely, May, the paddy field was in the leveling period to ensure that each paddy patch could store a certain amount of surface water. The surface vegetation cover of the paddy field was basically bare soil and water body. The land surface temperature (LST) of the paddy field was 27.73° in this month (Figure 6). June was the second month of the crop growing season. The transplanting stage (i.e., the process of paddy rice seedlings being transplanted and planted from the seedling raising shed to the land field) happened this month. The surface of the paddy field was mainly covered by water and sparse vegetation (i.e., rice seedlings). June was also the first month of summer in the study area, meaning the increase in solar radiation and temperature. The LST of the paddy field was 29.38°, which was higher than the May. Although the surface coverage in May was bare soil and water, the surface coverage in June was sparse vegetation and water. In this month, the increased radiation of the sun raised the land surface temperature. July and August were the third and fourth months of the growing season in the study area. It was also the hottest two months of the whole year in Northeast China, the second and last month of summer. In these two months, paddy plants began to grow rapidly. The surface of the paddy field was mainly covered by vegetation. Under the vegetation canopy, there was sufficient water, which came from surface water irrigation, groundwater exploitation, and rainwater, together to support the large water demand of paddy fields. Although in these two months, solar radiation has significantly increased land and air energy. The intense transpiration and evaporation of crops in paddy fields kept the surface temperature at a low level, with the LST of 27.01° and 25.62° in July and August, respectively. This means that in hot summer, paddy fields can provide a space with low surface temperature so as to improve the comfort of the living environment working in agricultural activity regions. September was the season for paddy crops to mature, the first month of autumn. At this stage, the water in the paddy field gradually dried up. The paddy field vegetation gradually changed from green to yellow, meaning that the evaporation and transpiration of water and vegetation were gradually weakening or even disappearing. Soil moisture gradually became the main factor affecting the surface temperature. The LST of the paddy field was 22.97°. October was the last month of the growing season and the season of rice paddy harvest. The yellow vegetation in the paddy field was harvested, and the bare soil gradually leaked out of the surface. Soil moisture was the main factor affecting the surface temperature. The LST of the paddy field was 14.36°, which was the lowest surface temperature in the growing season due to the climate in Northeast China began to cool in October.

### 3.4. Analysis of Cooling Effect of Paddy Field under the Different Scales in the Study Area

**Figure 7:**
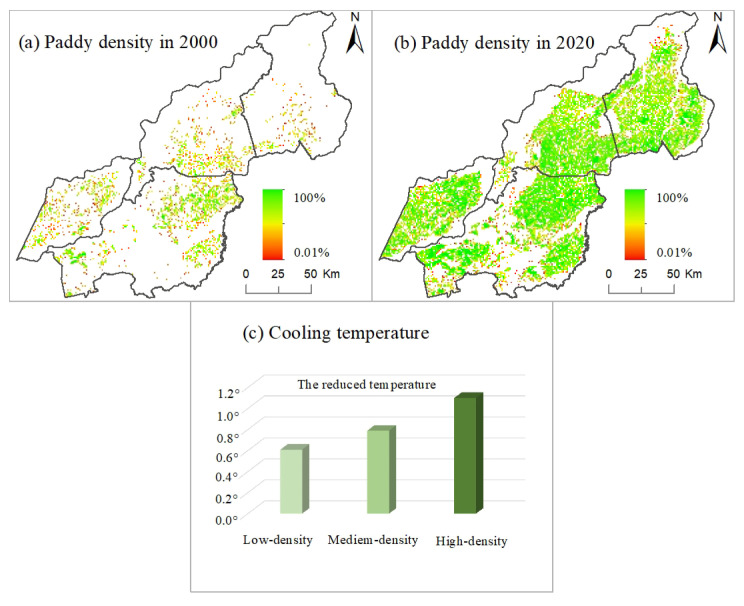
Effects of different paddy field sizes on land surface temperature.

Based on the paddy field grade map, we superimposed it on the surface temperature of the paddy field during the whole crop growing season to conduct the statistical analysis. The cooling surface temperature effect value of the low-density paddy field region was 0.61° (Figure 7). Further, we investigated the influence of the medium-density paddy field region on surface temperature and found the reduced temperature value was 0.79°. The data showed that the medium-density paddy field region brought a stronger cooling trend than the low-density paddy field region, with a different value of 0.18°. Similarly, we calculated the reduced temperature value in high-density paddy fields, in the same way, obtaining the value of 1.10°. The data displayed a stronger cooling effect in high-density paddy fields than in medium-density paddy fields, along with the different LST between both was 0.31°. Therefore, with the increase in paddy field planting scale, its impact on surface temperature was also gradually deepening, showing the cooling LST effect of paddy field expansion, and our survey gave the calculated value from 2000 to 2020 in the Northeastern Sanjiang Plain of China.

## 4. Discussion

### 4.1. The New Finding of Paddy Field Expansion from 2000 to 2020

This study found two new paddy field phenomena, one was the continuous expansion of paddy fields, and the other was the continuous northward migration of paddy fields from 2000 to 2020. 

For the continuous expansion of paddy fields, a time interval of 5-year dynamic monitoring of paddy fields from 2000 to 2020 was implemented in this study. A new paddy field phenomenon appeared in 2015–2020; that is, the expansion area of paddy field was only 252.68 km^2^ in this period, which was much lower than the previous period such as the 1446.74 km^2^, 3647.41 km^2^ and 3519.53 km^2^ in the periods of 2000–2005, 2005–2010 and 2010–2015, respectively. Therefore, our monitoring of paddy fields found that from 2015 to 2020, paddy fields in the study area entered a slow stage. Additionally, the reclamation rate was analyzed, with values of 10.66% in 2000 and 47.53% in 2020, and data showed that the paddy field area accounted for half of the whole study area in 2020. Theoretically, there was still space for the expansion of paddy field development. However, due to the requirements of forestry protection planning, grassland protection planning, natural resource protection planning and other relevant documents, such as the 14th five-year plan for urban development [42,43,44], the development space of paddy fields began to be limited from 2015 to 2020, leading to the rapid reduction of paddy field expansion speed at this stage.

For the migration trajectory of the paddy field, our finding was that the center of gravity of the paddy field continued to move northward in each period from 2000 to 2020. The straight-line migration distance of the paddy field gravity center reached 23,942.87 m (i.e., over 23 km^2^). Because our study area was located in the high latitude region of the Northern Hemisphere, which belonged to the cold temperate zone, the continuous northward migration and planting of rice needed to overcome the conditions of light and extremely low temperature [21,45]. A key factor of rice planting in cold temperate zone was to have sufficient light and accumulated temperature conditions. Especially in the extreme temperature years, such as the year of low temperature in early spring (i.e., generally in May and June) [46]. Extreme low temperatures often caused rice seedlings to freeze to death during this period [47]. With the continuous promotion of science and technology [48], research on low-temperature-resistant rice seedlings and short sunshine rice has been continuously promoted to the north, effectively overcoming the problems of extremely low temperature conditions in early spring and insufficient light in higher temperature regions and promoting the northward migration of paddy fields in Northeast China [23].

### 4.2. Influence of Paddy Field Expansion on the Comfort of Human Well-Being in Northeast China

Most of the current research on the comfort of human well-being mostly focused on the densely populated region, such as cities and urban agglomeration [49,50,51], to explore the impact of change in various index factors of surface radiation energy balance on the comfort of human settlements. However, less attention has been paid to agricultural activity areas with a sparse population, such as our research area, the paddy field planting center and the national grain production base. The spatiotemporal heterogeneity and expansion of paddy fields also changed the surface roughness of paddy fields and adjacent other land use types such as upland crops and grassland [52]. Different surface roughness of each land use type caused the horizontal disturbance of surface turbulence in the paddy field expansion region. Under the driving action of transpiration from paddy field vegetation and evaporation from water irrigation, horizontally disturbed airflow displayed the process of vertical rise, along with the upward transmission of energy in the paddy field region and forming the reduced temperature region. Therefore, the high-density paddy field region often formed a strong cooling land surface temperature effect in the growing season of crops in the study area through the water evaporation and rice paddy vegetation transpiration. Therefore, according to our investigation, the expansion of paddy fields formed the different low land temperature regions. The higher the density of paddy fields, the lower the temperature. From 2000 to 2020, the intense paddy field expansion area formed the cold island phenomenon or the cooling land surface temperature phenomenon in the paddy field region in the study area. In the hot summer in inland areas, such as the Northeast Sanjiang Plain, the ground temperature effect brought by the large-scale expansion of paddy fields can effectively reduce the temperature in paddy fields, which was conducive to improving the comfort of farmers in paddy field planting regions and making them feel cooler in hot summer, to avoid physical discomfort caused by high temperatures, such as heat stroke and human dehydration, considering that land surface temperature may be a factor affecting human well-being [53,54]. This comfort was beneficial not only to farmers but also to the tourists who enjoyed the natural scenery and agricultural engineering investigation, etc. Paddy field expansion served the well-being of human settlements in agricultural regions.

### 4.3. Comparative Analysis of Paddy Field Change and Its Cold Irrigation Effect in Different Regions

This study investigated the paddy field change and its cooling temperature effect through the use of remote sensing and land use expansion model in the cold temperate zone of the Northern Hemisphere. Spatiotemporal heterogeneity of paddy field expansion and its impact on temperature throughout the growing season were calculated. For the spatial distribution characteristics of paddy fields, previous literature displayed that there was a large number of paddy field planting regions in Northeast Asia, such as North Korea, Northern Japan, and Jilin and Liaoning provinces of Northeast China [21,23]. However, the development speed and scale of paddy fields in these areas lagged behind that of Sanjiang Plain [21,23]. Furthermore, contrary to the large-scale expansion of paddy fields in Sanjiang Plain, some literature reported that the planted scale of paddy fields in southeast coastal areas of China (i.e., the traditional paddy field planting region) continued to decline [36,55,56]. In addition to the comparison of paddy field changes, we also analyzed the difference in cooling temperature effect of different paddy fields from the perspective of low-, medium- and high-density regions. The temperature effect caused by irrigated paddy fields in Sanjiang plain, Northeast China, was calculated. In different regions, the differences in irrigation methods and types of irrigated crops often led to great differences in cooling temperature effects. In arid and semi-arid regions, drip irrigation and sprinkler irrigation were often used for farmland irrigation due to the relative lack of water resources [57]. The cooling temperature effect of irrigated farmland was weaker in arid and semi-arid regions than that of humid regions due to the fact that flood irrigation was often used in the farmland of humid regions, such as Sanmjiang Plain. In some humid regions, the cooling temperature effect of the irrigated farmland was even as high as 7° in the summer of July in some regions of the United States [33] compared with non-irrigated regions. This cooling value was also higher than that of our study area, Sanjiang Plain.

## 5. Conclusions

To reveal the new findings from 2000 to 2020, this study investigated the development level, quantitative characteristics, spatial evolution differences and the center of gravity migration trajectory of paddy fields and further explored the temperature characteristics of paddy fields in each month of the whole growing season and the cooling effect of different paddy fields on the human settlements from the perspective of the land surface temperature, using the method of artificial digitization, bilinear fitting and density segmentation, in the northeast part of Sanjiang Plain where the paddy field planting center in Northeast China and one of the national commodity grain production base. The main conclusions are below. 

(1) A sustained and acute paddy field expansion was monitored, with the total ranging area of paddy field from 2564.58 km^2^ in the year 2000 to 11,430.94 km^2^ in the year 2020, showing a total rate of growth of 345.72% and an average annual growth rate of 17.29%. Among the different stages with 5-year intervals from 2000 to 2020, the fastest increment of paddy field occurred in 2005–2010, with net change of 3647.41 km^2^. Correspondingly, the reclamation rate of paddy fields changed from 10.66% in 2000 to 47.53% in 2020, a total growth of 345.87%, showing the improved planting level of paddy fields.

(2) Paddy fields showed complex spatial variation characteristics in each period with 5-year intervals, and the center of gravity of paddy fields in the study area experienced a complex migration process, with a straight line of 23942.87 m from 2000 to 2020 and the direction offset of 27.20° from east to north. In this process, the center of gravity also moved from Fujin to Tongjiang.

(3) Throughout the growing season of paddy fields, the land surface temperature of paddy field in each month was 27.73°, 29.38°, 27.01°, 25.62° and 22.97°, indicating the maximum value of LST in June and the minimum in October. The LST of the paddy field was different each month. In hot summer, paddy fields can provide a space with low surface temperature to improve the comfort of the living environment in agricultural activity regions. Temperature reduction caused by low-, medium- and high-paddy field density regions from 2000 to 2020 was 0.61°, 0.79° and 1.10°, respectively, indicating a temperature effect of continuous cooling of 0.18° and 0.31° among the different paddy field density regions. This survey gave the new value from 2000 to 2020 in the study area.

Overall, to achieve the research goal of this study, we designed a theoretical framework including the contents of spatiotemporal dynamics, center of gravity migration, and the cooling effect of paddy fields. A collaborative research method that combined the human–computer interaction technology, gravity center model and spatial analysis was then established to gradually complete the studied framework. The contribution of the research content was to reveal the continuous expansion and northward migration of paddy fields and their cooling effect in the cold temperate zone of the high latitudes from 2000 to 2020. We looked forward to the designed theoretical framework, the collaborative research method and research findings on spatiotemporal characteristics, migration law and cooling effect of paddy fields in this study from 2000 to 2020 can provide the direct references or idea design for paddy field investigation in the cold temperate zone of the high latitudes and the indirect comparison of research on irrigated paddy fields in other regions.

## Figures and Tables

**Table 1:** Paddy land area and corresponding reclamation rate in each administrative region in the years 2000, 2005, 2010, 2015 and 2020 (area: km^2^, reclamation ratio: %).

	Fuyuan City		Tongjiang City		Suibin City		Fujin City	
Year	Paddy	Reclamation	Paddy	Reclamation	Paddy	Reclamation	Paddy	Reclamation
	Area	Ratio	Area	Ratio	Area	Ratio	Rrea	Ratio
2000	223.35	3.67	586.14	9.60	528.32	15.71	1226.86	14.45
2005	475.15	7.80	1019.24	16.69	693.87	20.63	1823.19	21.48
2010	1634.03	26.82	2000.37	32.76	1317.44	39.17	2707.15	31.89
2015	3043.35	49.96	2563.38	41.99	1749.59	52.02	3821.94	45.03
2020	3103.40	50.95	2569.24	42.08	1775.33	52.78	3982.97	46.92

## Data Availability

The data presented in this study are available on request from the corresponding author.
